# Cyclooxygenase-2 and human epidermal growth factor receptor type 2 (HER-2) expression simultaneously in invasive and *in situ* breast ductal carcinoma

**DOI:** 10.1590/S1516-31802011000600002

**Published:** 2011-12-01

**Authors:** Adrienne Pratti Lucarelli, Maria Marta Martins, Wagner Montor, Vilmar Oliveira, Maria Antonieta Longo Galvão, Sebastião Piato

**Affiliations:** I MD, PhD. Instructor professor, Mastology Unit of the Department of Gynecology and Obstetrics, Santa Casa de Misericórdia de São Paulo Hospital, Faculdade de Ciências Médicas da Santa Casa de São Paulo (FCMSCSP), São Paulo, Brazil.; II PhD. Professor, Department of Biochemistry, Faculdade de Ciências Médicas da Santa Casa de São Paulo (FCMSCSP), São Paulo, Brazil.; III PhD. Instructor professor, Department of Pathology, Faculdade de Ciências Médicas da Santa Casa de São Paulo (FCMSCSP), São Paulo, Brazil.; IV MD, PhD. Assistant professor, Department of Pathology, Faculdade de Ciências Médicas da Santa Casa de São Paulo (FCMSCSP), São Paulo, Brazil.; V MD, PhD. Full professor, Mastology Unit of the Department of Gynecology and Obstetrics, Santa Casa de Misericórdia de São Paulo Hospital, Faculdade de Ciências Médicas da Santa Casa de São Paulo (FCMSCSP), São Paulo, Brazil.

**Keywords:** Cyclooxygenase 2, Receptor, erbB-2, Breast neoplasms, Carcinoma, intraductal, noninfiltrating, Carcinoma, ductal, breast, Ciclooxigenase 2, Receptor erbB-2, Neoplasias da mama, Carcinoma intraductal não infiltrante, Carcinoma ductal de mama

## Abstract

**CONTEXT AND OBJECTIVE:**

Cyclooxygenase-2 (COX-2) and human epidermal growth factor receptor type 2 (HER-2) are associated with tumorigenesis. Studies have shown that HER-2 can regulate COX-2 expression. The aim of this study was to evaluate the correlation between COX-2 and HER-2 expression in normal breast epithelium and in ductal carcinoma *in situ* (DCIS) and invasive ductal carcinoma (IDC) present in the same breast.

**DESIGN AND SETTING:**

Cross-sectional study at the Mastology Unit of the Department of Gynecology and Obstetrics, Santa Casa de Misericórdia de São Paulo Hospital.

**METHODS:**

COX-2 and HER-2 were detected using immunohistochemistry on 100 tissue fragments. HER-2 ≥ +2 was subjected to fluorescence in situ hybridization (FISH).

**RESULTS:**

COX-2 expression was detected in 87%, 85% and 75% of IDC, DCIS and normal epithelium, respectively. HER-2 expression was detected in 34% of IDC and 34% of DCIS. COX-2 in DCIS correlated with HER-2 in IDC (P = 0.049) and DCIS (P = 0.049). COX-2 in normal epithelium correlated with HER-2 in IDC (P = 0.046) and DCIS (P = 0.046). COX-2 in IDC was not associated with HER-2 (P = 0.235). Comparison between COX-2 and HER-2 in DCIS showed that there was a statistically significant difference with regard to nuclear grades II and III and presence of comedonecrosis (P < 0.001). In IDC, there was significant expression with nuclear grades II and III and histological grade II (P < 0.001).

**CONCLUSIONS:**

Our findings provide evidence that HER-2 and COX-2 regulate each other.

## RESUMO

**CONTEXTO E OBJETIVO:**

Ciclo-oxigenase (COX-2) e receptor tipo 2 do fator de crescimento epidérmico humano (HER-2) estão associados com tumorigênese. Estudos mostraram que HER-2 pode regular a expressão de COX-2. O objetivo deste estudo foi avaliar a correlação entre expressão da COX-2 e HER-2 no epitélio normal de mama, no carcinoma ductal *in situ* (DCIS) e carcinoma ductal invasivo (IDC) presentes na mesma mama.

**TIPO DE ESTUDO E LOCAL:**

Estudo transversal na clínica de Mastologia do Departamento de Obstetrícia e Ginecologia do Hospital da Santa Casa de Misericórdia de São Paulo.

**MÉTODOS:**

A detecção da COX-2 e HER-2 foi realizada por imunoistoquímica em 100 fragmentos teciduais. HER-2 ≥ +2 foi submetido a hibridização fluorescente *in situ* (FISH).

**RESULTADOS:**

Expressão de COX-2 foi detectada em 87%, 85% e 75% dos IDC, DCIS e epitélio normal, respectivamente. Expressão de HER-2 foi detectada em 34% dos IDC e 34% de DCIS. COX-2 em DCIS correlacionou-se com HER-2 em IDC (P = 0,049) e DCIS (P = 0,049). COX-2 no epitélio normal correlacionou-se com HER-2 em IDC (P = 0,046) e DCIS (P = 0,046). COX-2 no IDC não foi associada com HER-2 (P = 0,235). Quando comparado COX-2 com HER-2 em DCIS houve diferença estatisticamente significante com relação ao grau nuclear II e III e presença de comedonecrose (P < 0,001) e no IDC, houve expressão significativa no grau nuclear II e III e histológico II (P < 0,001).

**CONCLUSÕES:**

Nossos achados mostram evidências que HER-2 e COX-2 se autorregulam.

## INTRODUCTION

Prostaglandins are known to participate in multiple physiological and pathological processes, including wound healing, cardiovascular disease, inflammation and the development and growth of malignant tumors.^1^ Cyclooxygenase-2 (COX-2), the inducible isoform of prostaglandin H synthase, has been implicated in the origin of a variety of human cancers, including breast, colon, lung, gastric, skin, endometrial, ovarian and esophageal adenocarcinomas.^2,3^ COX-2 seems to be involved in the processes of malignant transformation and tumor progression by affecting cell proliferation, mitosis, cell adhesion, apoptosis, immune surveillance, angiogenesis and formation of carcinogenic metabolites such as malondialdehyde.^4,5^

Breast cancer is the most common cancer in women and is the second biggest cause of cancer-related mortality in the world.^6^ Epidemiological studies have suggested that regular use of non-steroidal anti-inflammatory drugs (NSAIDs) may have some protective effect against breast cancer.^7^ Significantly reduced risk of human breast cancer relating to intake of selective COX-2 inhibitors has been reported.^8^

COX-2 expression is induced by pro-inflammatory cytokines, tumor promoters, growth factors and viral transformation.^9,10^ How COX-2 overexpression results in tumorigenesis and how COX-2 selective agents mediate chemopreventive effects are issues that still need further clarification.

Another important protein, closely related to breast cancer, is human epidermal growth factor receptor type 2 (HER-2), one of the four receptors belonging to the HER family.^11,12^ This receptor presents proliferation-stimulating responses upon ligand binding.^13^

HER-2 overexpression often results from increased c-erb-b2 gene activation.^14,15^ Its overexpression becomes more important when this protein forms heterodimers with other HER family receptors, such as human epidermal growth factor receptor type 3 (HER-3). This leads to hyperactive cell signaling pathways and the emergence of a situation of uncontrolled cell proliferation, thus culminating in tumorigenesis.^16^

Overexpression of this protein in breast cancer has been associated with disease aggressiveness, a more reserved prognosis, absence of estrogen and progesterone receptors and resistance to hormonal therapy and chemotherapy based on cyclophosphamide, methotrexate and fluorouracil.^17^

Studies have reported that HER-2 can regulate COX-2 expression.^18^ Increased levels of COX-2 messenger RNA (mRNA), proteins and, consequently, prostaglandin E_2_ synthesis have been detected in HER-2 transformed human mammary epithelial cells, compared with the non-transformed partner cell line. In this case, it was observed that HER-2 stimulated COX-2 transcription via Ras → Raf → mitogen-activated protein kinase (MAPK).^18^ Supporting this hypothesis, studies have demonstrated COX-2 expression in HER-2 positive invasive breast cancer samples.^19,20^

Research to test whether improved chemopreventive efficacy could be achieved by combining submaximal doses of selective COX-2 inhibitors and selective retinoid for preventing HER-2 mammary tumorigenesis has shown that selective COX-2 inhibitor and retinoid in combination mediate greater-than-additive suppression of mammary tumor formation in HER-2 transgenic mice. Furthermore, parallel decreases in mammary aromatase activity have been observed. The combination of celecoxib and retinoid potently suppressed mammary aromatase activity (by 56%), compared with controls, thus suggesting that aromatase modulation may contribute towards mediating tumor suppression.^21^

Many studies have already analyzed HER-2 and COX-2 expression in ductal carcinoma *in situ* and invasive ductal carcinoma, but most of these studies were carried out on samples from different women, thereby restricting the usefulness of these data for studying tumor progression.

## OBJECTIVE

The aim of this study was to evaluate the correlation between COX-2 and HER-2 expression in ductal carcinoma *in situ* (DCIS) and invasive ductal carcinoma (IDC) from the same breast as well as in normal epithelium. Additionally, we also correlated protein expression with nuclear grade, histological grade, presence or absence of comedonecrosis, tumor size (smaller or equal to 2 cm or greater than 2 cm) and age at time of diagnosis (younger than 50 years or 50 years and over).

## METHODS

### Sample collection

This study was carried out by analyzing 150 tissue samples obtained from 150 patients who underwent breast cancer surgical treatment at the Santa Casa de Misericórdia de São Paulo Hospital between June 2002 and July 2008. Fifty cases were excluded as ineligible for the study: in 30 of these cases, the specimens presented insufficient amounts of invasive ductal carcinoma (IDC), ductal carcinoma *in situ* (DCIS) and normal epithelium; and 20 specimens did not show invasive tumor and ductal carcinoma *in situ* in the same paraffin block. Only 100 samples were usable in this longitudinal retrospective study.

The patients’ mean age was 52 years (range: 31-85 years). None of the patients had received chemotherapy, hormonal treatment or radiation therapy before surgery, or any non-steroidal anti-inflammatory drugs for the 15 days preceding the procedure.

The hematoxylin and eosin slides for all cases were reviewed. The nuclear grade and presence or absence of comedonecrosis in DCIS and the nuclear grade and histological type in IDC were determined.^22,23^

### Tissue preparation and immunohistochemistry

Sections of 3 μm in thickness from specimens in formalin-fixed, paraffin-embedded blocks were prepared stepwise, with deparaffinization in xylene and rehydration in descending alcohols. Antigens were retrieved using a microwave oven (four times 5 min at 700 W in 10 mM sodium citrate buffer, pH 6.0). The slides were immersed in 0.3% hydrogen peroxide in methanol for 30 minutes to block endogenous peroxidase activity and then in blocking solution [1.5:100 normal horse serum in phosphate-buffered saline (PBS) for blocking nonspecific binding sites.

COX-2 immunostaining was performed using specific polyclonal antibody (3362-100, Biovision Research Products Co., Mountain View, California, United States) at a dilution of 1:70 (15 μg/ml) for 18 hours. HER-2 immunostaining was performed using mouse monoclonal anti-human HER-2 (clone CB11, Dako A/S, Copenhagen, Denmark) at a dilution of 1:150 for 18 hours.

The slides were then rinsed in PBS, and the biotinylated secondary antibody was applied at room temperature for 30 minutes (Dako Corp., California, United States). The slides were incubated in streptavidin-peroxidase at 37 °C for 30 minutes. The specimens were rinsed in 0.005% Tween-20 in PBS and then incubated with the 3.3′- diaminobenzidine chromogenic substrate for five minutes. The sections were counterstained in Meyer's hematoxylin and were then mounted.

### HER-2 fluorescence in situ hybridization (FISH) analysis

Tumors that were scored +2 for membranous staining using the Dako HercepTest kit were subjected to FISH analysis using the Vysis PathVysion kit, in accordance with the manufacturer's instructions. The Vysis kit incorporates a control probe for chromosome 17, as well as the test probe for the *HER-2* gene. In brief, 4-μm paraffin-embedded sections were dewaxed, taken to absolute ethanol, and air dried. They were then placed in 0.2 M HCl at room temperature for 20 minutes and in pretreatment solution at 80 °C for 30 minutes, and then underwent proteolytic digestion at 37 °C for 25 minutes. The sections were then denatured in formamide at 72 °C for 5 minutes before incubation in the PathVysion HER-2/17 probe overnight in the dark at 37 °C. The following day, the sections were washed in post-hybridization buffer at 72 °C for 2 minutes, air dried in the dark, and then mounted in 4’,6-diamidino-2-phenylindole.

### Evaluation of COX-2 and HER-2 staining

COX-2 and HER-2 immunohistochemical staining were evaluated and scored independently and blindly by two investigators.

**Figure 1:**
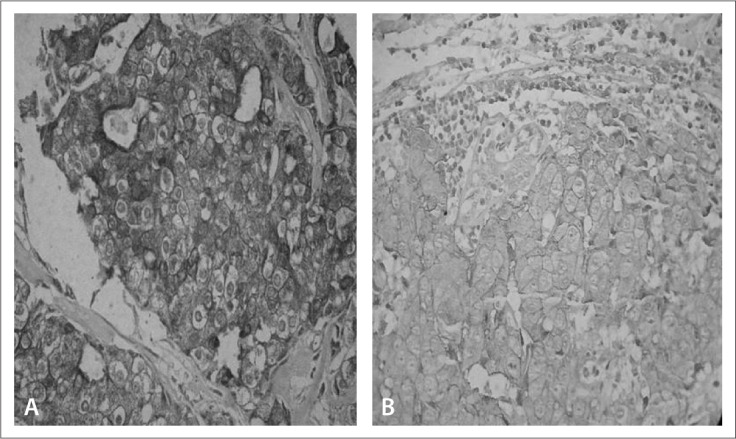
(A) Invasive ductal carcinoma (IDC) showing score 3 cyclooxygenase-2 (COX-2) expression. (B) IDC showing score 3 HER-2 expression.

For COX-2, we used the same criteria adopted by Ristimäki et al.:^19^ score 0, no staining; score 1, weak diffuse cytoplasmic staining (may contain stronger intensity in less than 10% of cells); score 2, moderate to strong granular cytoplasmic staining in 10-90% of cells; and score 3, over 90% of cells stained with strong intensity. COX-2 expression was considered positive with scores of 2 or 3 and negative with scores of 0 or 1 (Figure 1a).

HER-2 expression analysis was performed as recommended by the HercepTest scoring guidelines: score 0, no staining or < 10% membrane staining; score +1, partial membrane staining in > 10% of the tumor cells; score +2, weak or moderately complete membrane staining in > 10% of the tumor cells; and score +3, strong complete membrane staining in > 10% of the tumor cells. For HER-2, tumors that exhibited membrane staining of +3 intensity or that were +2 but showed gene amplification by means of FISH analysis were considered positive (Figure 1b).

Sections were scored by one investigator and subjected to review by a second investigator.

### Statistical analysis

The correlation between COX-2 and HER-2 was analyzed using Spearman's rank coefficient. The Kruskal-Wallis test was used to analyze nuclear grade and histological grade, and the Mann-Whitney test was used to check for the presence or absence of comedonecrosis. The chi-square test was used to analyze the ages and tumor size groups. P-values < 0.05 were considered statistically significant. All statistical analyses were performed using the Statistical Package for the Social Sciences software, version 13.0 (SPSS Inc., Chicago, Illinois, United States).

## RESULTS

### COX-2 expression

One hundred cases were evaluated for immunoreactivity with anti-COX-2 antibodies. COX-2 immunoreactivity was characteristically cytoplasmic and granular. COX-2 was positively detected at frequencies of 87%, 85% and 75% in IDC, DCIS and normal epithelium respectively.

### HER-2 expression

HER-2 was analyzed by means of immunohistochemistry in one hundred cases. Membrane staining of +2 intensity was detected in seven cases, and these were subjected to FISH analysis for HER-2 gene amplification. None of the seven tumors with +2 membrane staining showed chromosomal HER-2 amplification. Membrane staining of +3 intensity was detected in IDC and DCIS at a frequency of 34% for both, and no staining was found in the sampled areas of normal epithelium. Thus, 34% of the tumors were considered to be HER-2 positive, both in IDC and in DCIS.

### Correlation between COX-2 and HER-2 expression

COX-2 expression in DCIS correlated with high HER-2 expression in IDC (P = 0.049) and DCIS (P = 0.049). COX-2 expression in normal epithelium correlated with high HER-2 in IDC (P = 0.046) and DCIS (P = 0.046). Elevated COX-2 expression in IDC was not associated with HER-2 expression in IDC, DCIS or normal epithelium (P = 0.235) (Table 1).

**Table 1.** Association of expression of human epidermal growth factor receptor type 2 (HER-2) and cyclooxygenase-2 (COX-2) in non-neoplastic ducts, ductal carcinoma *in situ* (DCIS) and invasive ductal carcinoma (IDC) of the same breast and statistical analysis by the Spearman correlation (R)

Histological tissue	Statistical analysis	HER-2 IDC	HER-2 DCIS	HER-2 normal epithelium
**COX-2 IDC**	Correlation coefficient	0.177	0.177	-
	Significant (P)	0.235	0.235	-
	n	100	100	100
**COX-2 DCIS**	Correlation coefficient	0.289	0.289	-
	Significant (P)	**0.049**	**0.049**	-
	n	100	100	100
**COX-2 normal epithelium**	Correlation coefficient	0.293	0.293	-
	Significant (P)	**0.046**	**0.046**	-
	n	100	100	100

Comparison of high levels of COX-2 expression and high levels of HER-2 expression in DCIS, according to different nuclear grades and the presence of comedonecrosis, showed that there was statistically significant expression between the two proteins with regard to nuclear grades II and III and the presence of comedonecrosis (P < 0.001). When focusing on IDC, we found significantly positive expression with nuclear grades II and III and histological grade II between COX-2 and HER-2 (P < 0.001).

We found in tumors of sizes less than or equal to 2 cm that COX-2 expression significantly correlated with HER-2 in IDC, DCIS and normal epithelium (P < 0.03). In tumors larger than 2 cm, there was also significantly higher expression of COX-2 and HER-2 in IDC, DCIS and normal epithelium (P < 0.001) (Table 2 and Table 3).

**Table 2.** Expression of cyclooxygenase-2 and human epidermal growth factor receptor type 2 (HER-2) in 100 cases in tumors of sizes less than or equal to 2 cm and statistical analysis using the chi-square test

Histological tissue	Cyclooxygenase-2		HER-2		Significance (P)
	Tumors ≤ 2 cm		Tumors ≤ 2 cm		
	Cases	%	Cases	%	
**Invasive ductal carcinoma**					
Positive	23	92%	8	33.33%	0.003
Negative	2	8%	17	66.67%	
N	25	100%	25	100%	
**Ductal carcinoma *in situ***					
Positive	23	92%	8	33.33%	0.003
Negative	2	8%	17	66.67%	
N	25	100%	25	100%	
**Normal epithelium**					
Positive	21	84%	0	0.00%	< 0.001		
Negative	4	16%	25	100%
N	25	100%	25	100%

**Table 3.** Expression of cyclooxygenase-2 (COX-2) and human epidermal growth factor receptor type 2 (HER-2) in 100 cases, in tumors larger than 2 cm and statistical analysis using the chi-square test

Histological tissue	COX-2		HER-2		Significance (P)
	Tumors > 2 cm		Tumors > 2 cm		
	Cases	%	Cases	%	
**Invasive ductal carcinoma**					
Positive	66	88%	26	34.66%	< 0.001
Negative	9	12%	49	65.33%	
N	75	100%	35	100%	
**Ductal carcinoma *in situ***					
Positive	65	86.66%	26	34.66%	< 0.001
Negative	10	13.33%	49	65.33%	
N	75	100%	75	100%	
**Normal epithelium**					
Positive	56	75.66%	0	0.00%	< 0.001
Negative	19	25.33%	75	100%	
N	75	100%	75	100%	

In spite of age, for patients aged 50 years or over, COX-2 and HER-2 expression was statistically significant in IDC, DCIS and normal epithelium (P < 0.001). In patients less than 50 years of age, again, COX-2 and HER-2 expression was statistically significant in IDC and DCIS (P = 0.005), and in normal epithelium (P < 0.001) (Table 4 and Table 5).

**Table 4.** Expression of cyclooxygenase-2 (COX-2) and human epidermal growth factor receptor type 2 (HER-2) in 100 patients aged 50 years or over and statistical analysis using the chi-square test

Histological tissue	COX-2		HER-2		Significance (P)
	≥ 50 years		≥ 50 years		
	Cases	%	Cases	%	
**Invasive ductal carcinoma**					
Positive	58	90.66%	21	32.81%	< 0.001
Negative	6	9.44%	43	67.18%	
N	64	100%	64	100%	
**Ductal carcinoma *in situ***					
Positive	55	85.99%	21	32.81%	< 0.001
Negative	9	14.07%	43	67.18%	
N	64	100%	64	100%	
**Normal epithelium**					
Positive	49	76.56%	0	0.00%	< 0.001
Negative	15	23.43%	64	100%	
N	64	100%	64	100%	

**Table 5.** Expression of cyclooxygenase-2 (COX-2) and human epidermal growth factor receptor type 2 (HER-2) in 100 patients less than 50 years of age and statistical analysis using the chi-square test

Histological tissue	COX-2		HER-2		Significance (P)
	< 50 years		< 50 years		
	Cases	%	Cases	%	
**Invasive ductal carcinoma**					
Positive	30	83.33%	13	36.11%	0.005
Negative	6	16.66%	23	63.88%	
N	36	100%	36	100%	
**Ductal carcinoma *in situ***					
Positive	30	83.33%	13	36.11%	0.005
Negative	6	16.66%	23	63.88%	
N	36	100%	36	100%	
**Normal epithelium**					
Positive	25	69.44%	0	0.00%	< 0.001
Negative	11	30.55%	367	100%	
N	36	100%	36	100%	

## DISCUSSION

Research studies examining the tumor microenvironment are providing information on the molecular mechanisms behind the role played by inflammation in initiating or promoting a wide range of cancers. This has important implications for prevention, monitoring and treatment. In this manner, the enzyme COX-2 is emerging as an important biological marker, among other agents.^24,25^

The exact mechanisms through which COX-2 is upregulated in breast cancer are not completely understood yet, but one possible explanation is that the expression of this enzyme in breast cancer cells is more active than in normal tissue because of intrinsic mechanisms. Such mechanisms would include the association between COX-2 expression and HER-2.^18^ The activation of the HER-2/HER-3 pathways in colon cancer cell lines can induce COX-2 mRNA and protein as well as prostaglandin E_2_ (PGE_2_) biosynthesis.^26^

Recent studies exploring the relationship between tumor progression and nuclear factor-kappaB (NFkB) activation have brought an important molecular link between tumor progression and inflammation. In some tumors, contrary to what happens in normal tissues, NFkB activation due to inflammation renders cells resistant to apoptosis,^27-29^ which can at least partially be reverted through the use of COX inhibitors. Studies have shown that it is even possible to revert tamoxifen resistance in specific breast cancer cells through the use of NFkB inhibitors,^30^ and to observe a direct correlation between NFkB activity and HER-2/neu expression, thereby suggesting a possible role for COX activity in tumor progression.^26^

In breast cancer cases, the constitutive activity of NFkB causes losses in estrogen receptor and resistance antibody-based therapies, through signaling events downstream of HER-2. One of the key targets of NFkB is inducible cyclooxygenase, i.e. the enzyme responsible for the conversion of arachidonic acid to PGE_2_. Conjugated linoleic acid in cell cultures has been shown to inhibit HER-2 and COX-2 protein expression, and it has been found that reduction of HER-2 correlated with a reduction in PGE_2_ synthesis.^26^

Dillon et al. studied a microarray comprising tumors from 560 patients, with analysis on HER-2 and COX-2 expression. The expression of the markers examined was analyzed in relation to classic clinicopathological parameters, in the presence and absence of tamoxifen. HER-2 and COX-2 were both found to be predictive of poor disease-free survival in patients on endocrine treatment. Positive COX-2 status among the patients was found to be predictive of adverse effects from tamoxifen. These clinical ex vivo data are consistent with molecular observations that HER-2 can regulate COX-2 expression through direct transcriptional mechanisms. COX-2 expression correlates with disease progression on endocrine treatment. This study supports a role for COX-2 as a predictor of adverse effects from tamoxifen in breast cancer patients.^31^

Other authors studied tissue samples from 64 women with breast cancer to examine the levels of transcripts of COX-2 and 12-lipoxygenase and to compare between the expressions of the two enzymes. They found that 41% of the cases examined exhibited overexpression of HER-2 and 61.5% of them showed overexpression of COX-2; while in HER-2 negative cases, COX-2 overexpression was detected in 36.8% of them. However, the statistical analysis came close to a significant relationship (P = 0.0747) between COX-2 expression and HER-2 patient status. For 12-lipoxygenase, 38% cancer specimens showed overexpression. Forty-one percent of the cases examined exhibited overexpression of HER-2 and 61.5% of them showed overexpression of 12-lipoxygenase; while in HER-2 negative cases, 12-lipoxygenase overexpression was detected in 63.2% of them. However, the statistical analysis did not show any significant relationship (P = 1.0) between 12-lipoxygenase expression and HER-2 patient status.^32^

COX-2 was expressed positively in IDC and DCIS in 87 (87%) and 85 (85%) of the cases respectively, similarly to other reports.^21,24,33^ The data of the present study showed that the COX-2 expression in DCIS and in IDC was frequently higher. Boland et al.^24^ observed that when the two types of tumor component (IDC and DCIS) were present in the same surgical specimen, the rate of positivity in the invasive component was greater than that observed in breast tumors that only showed invasive cancer. The expression of COX-2 in the *in situ* component possibly upregulates the presence of the enzyme in the invasive carcinoma, since its levels are usually higher in DCIS. In studies on normal epithelium adjacent to breast cancer, Half et al.^20^ and Shim et al.^34^ found positive COX-2 expression in 81% and 85% of the cases, respectively, and our result of 75% is in agreement with their data. It appears, therefore, that the presence of COX-2 in normal epithelium must play a preponderant role in the paracrine regulatory mechanisms of this enzyme in breast cancer, thus showing a possible role for local inflammation in tumor progression.

COX-2 expression in DCIS correlated with tumor aggressiveness, since the expression level was found to be higher in nuclear grade III tumors and in those presenting comedonecrosis. With regard to IDC, COX-2 expression was also higher in nuclear grade III tumors. Our data corroborate the findings reported by other authors.^32,35^

In parallel, we studied HER-2 and found that 34% of the IDC and DCIS cases were positive for this protein. Our results are concordant with a variety of findings described for other invasive carcinomas.^28-30^

We observed that when there were two tumor components (DCIS and IDC) in the same paraffin block, the rate of positivity for HER-2 seemed to be the same for the two regions (34% and 34%). As expected, analysis on normal epithelium did not show HER-2 expression through immunohistochemical staining.

We found that HER-2 in DCIS had greater expression in nuclear grade III tumors and in those presenting comedonecrosis. Moreover, HER-2 expression in IDC was statistically significant in relation to nuclear grade III and histological grade. Also with regard to IDC, COX-2 expression was higher in nuclear grade III tumors. Our results suggest that these were situations of tumor aggressiveness, since the expression level for HER-2 was higher. Similar data have also been described by other authors.^11,12,14,15^

Comparison of high levels of COX-2 expression and high levels of HER-2 expression in DCIS, according to different nuclear grades and the presence of comedonecrosis, showed that there was statistically significant expression between the two proteins with regard to nuclear grades II and III and the presence of comedonecrosis (P < 0.001). When focusing on IDC, we found significantly positive expression with nuclear grades II and III and histological grade II between COX-2 and HER-2 (P < 0.001).

We found in tumors of sizes less than or equal to 2 cm that COX-2 expression significantly correlated with HER-2 in IDC, DCIS and normal epithelium (P < 0.03). In tumors larger than 2 cm, there was also significantly higher expression of COX-2 and HER-2 in IDC, DCIS and normal epithelium (P < 0.001).

In spite of age, for patients aged 50 years or over, COX-2 and HER-2 expression was statistically significant in IDC, DCIS and normal epithelium (P < 0.001). In patients less than 50 years of age, again, COX-2 and HER-2 expression was statistically significant in IDC and DCIS (P = 0.005), and in normal epithelium (P < 0.001).

Several studies have addressed the expression of HER-2 and COX-2 in breast cancer. The exact molecular mechanism behind the coexpression of these two proteins is not completely understood yet, although some insights exist.^26,31,32^ We compared the expression of COX-2 and HER-2 in breast cancers by means of immunohistochemistry and FISH. Our observations indicate that there is a highly significant correlation between COX-2 expression and HER-2 expression, both in IDC and in DCIS. In addition, we studied the expression of this protein in non-neoplastic ducts (normal epithelium) and we did not obtain any statistical correlation between COX-2 and HER-2 expression.

We hypothesize that higher COX-2 expression correlates with higher HER-2 expression. This hypothesis is consistent with the findings from this study. Our results are in agreement with those obtained by Boland et al.,^24^ Perrone et al.,^36^ Subbaramaiah et al.,^18^ Benoit et al.,^37^ Cho et al.,^38^ Dillon et al.^31^ and Mohamed et al.^32^

One current point of discussion is that effects linked with the expression level of this protein, such as nuclear grade, histological grade, presence of comedonecrosis, tumor size and age at the time of diagnosis are also due to independent mechanisms for COX-2 and HER-2. In our study, high levels of COX-2 protein were reported more frequently in breast cancer cases with overexpressed of HER-2 when DCIS and IDC were both present in the same breast. The fact that our analysis was performed in simultaneous tumors might account for the higher levels of expression found; consequently, a direct relationship between COX-2 and HER-2 might be more readily observed. In addition, the level of HER-2 expression may correlate with activity levels and the ability to induce COX-2.^21^ The mechanisms through which COX-2 and HER-2 expression is upregulated in these processes are unclear, and many pathways and factors are known to be involved in carcinogenesis.

## CONCLUSIONS

From analysis of our results, we could confirm that there is a positive correlation between COX-2 and HER-2 in DCIS and IDC. Breast cancer cases with high levels COX-2 expression showed the presence of HER-2 expression in DCIS and CDI and these two enzymes did not show expression in normal ephitelium.
